# A remarkable basic hypergeometric identity

**DOI:** 10.1007/s11139-024-00994-4

**Published:** 2025-01-31

**Authors:** Christian Krattenthaler, Wadim Zudilin

**Affiliations:** 1 Fakultät für Mathematik, Universität Wien, Oskar-Morgenstern-Platz 1, 1090 Vienna, Austria; 2 Department of Mathematics, IMAPP, Radboud University, PO Box 9010, 6500 GL Nijmegen, The Netherlands

**Keywords:** Hypergeometric series, Root of unity, Telescoping, 11A07, 11B65, 11R18, 33D15, 33F10

## Abstract

We give a closed form for *quotients* of truncated basic hypergeometric series where the base *q* is evaluated at roots of unity.

We set $(a;q)_n=(1-a)(1-aq)\cdots (1-aq^{n-1})$ to be the *q*-shifted factorial (*q*-Pochhammer symbol), with its multiple version

$$\begin{aligned} (a_1,\dots ,a_m;q)_k=\prod _{j=1}^m(a_j;q)_k. \end{aligned}$$

An investigation of supercongruences for truncated $_4F_3$ hypergeometric sums in [6] revealed an interesting structure for their *q*-counterparts at roots of unity. In order to state the corresponding identity, we introduce the sum

1 $$\begin{aligned}  &   F_n(a,q;\ell _1,\ell _2) =\sum _{k=0}^{n-1}f_k(a,q;\ell _1,\ell _2), \nonumber \\  &   \text {where}\; f_k(a,q;\ell _1,\ell _2)=\frac{(q^{\ell _1}a,q^{1-\ell _1}a,q^{\ell _2}a,q^{1-\ell _2}a;q)_k}{(qa;q)_k^4}\,q^k, \end{aligned}$$

and the product

2 $$\begin{aligned} G(a,q;\ell _1,\ell _2) =\frac{\prod _{j=0}^{\ell _1-1}(a-q^j)\cdot \prod _{j=0}^{\ell _2-1}(a-q^j)}{\prod _{j=0}^{\ell _1-1}(1-q^ja)\cdot \prod _{j=0}^{\ell _2-1}(1-q^ja)}. \end{aligned}$$

Here $\ell _1,\ell _2$ are integers; they are assumed to be non-negative in (2). If one (or both) of $\ell _1$ and $\ell _2$ are zero, empty products have to be interpreted as 1.

## Theorem 1

For $\zeta =\zeta _n$ a primitive *n*th root of unity and $\ell _1,\ell _2\ge 0$ integers, define $F_n(a,\zeta )=F_n(a,\zeta ;\ell _1,\ell _2)$. Then

3 $$\begin{aligned} \frac{F_n(a,\zeta )}{F_n(1,\zeta )} =\frac{n^2a^{n-1}}{(1+a+a^2+\dots +a^{n-1})^2}\,G(a,\zeta ;\ell _1,\ell _2). \end{aligned}$$

## Remarks

(1) As a corollary, we obtain

$$\begin{aligned} \frac{F_n(a,\zeta )F_n(1/a,\zeta )}{F_n(1,\zeta )^2}=\bigg (\frac{na^{(n-1)/2}}{1+a+a^2+\dots +a^{n-1}}\bigg )^4 \end{aligned}$$

independent of the choice of the primitive *n*th root of unity; this formula is a result stated without proof in [6].

(2) We may extend the definition of the product to cases where the upper bound on the product index is less than the lower bound by

4 $$\begin{aligned} \prod _{k=M} ^{N-1}\operatorname {Expr}(k)={\left\{ \begin{array}{ll} \prod _{k=M} ^{N-1} \operatorname {Expr}(k)& N>M,\\ 1& N=M\\ 1\Big /\prod _{k=N} ^{M-1}\operatorname {Expr}(k)& N<M.\end{array}\right. } \end{aligned}$$

With this convention, we may observe that

$$\begin{aligned} G(a,q;-\ell _1,\ell _2)=G(a,q;\ell _1+1,\ell _2), \end{aligned}$$

and the analogous equation if the roles of $\ell _1$ and $\ell _2$ are interchanged. Since, trivially, $F_n(a,q;\ell _1,\ell _2)$ satisfies the same identities, we see that the restriction on $\ell _1$ and $\ell _2$ in Theorem 1 can be dropped when assuming the convention in (4).

The identity in Theorem 1 is somewhat unusual when compared to other evaluations of basic hypergeometric series, when those are expressed in ‘closed form’ — a product or a finite linear combination of products. We were not able to produce a closed-form evaluation for the sum $F_n(a,\zeta )$ itself, only for its renormalisation $F_n(a,\zeta )/F_n(1,\zeta )$ which is not a sum but a *quotient* of sums. Identity (3) connects visually — though not explicitly — with known evaluations involving the cyclic quantum dilogarithm [2] (see also [1, Appendix A]); it also shares similarities with identities obtained in [4].

We prove Theorem 1 by constructing explicit difference equations for the sums $F_n(a,\zeta ;\ell _1,\ell _2)$ with respect to the $\ell$-parameters, separately for the cases $\ell _1\ne \ell _2$ and $\ell _1=\ell _2$, by demonstrating that the expressions $G(a,\zeta ;\ell _1,\ell _2)F_n(1,\zeta ;\ell _1,\ell _2)$ satisfy the same equations, and by finally verifying the proportionality of these two solutions for a few starting values (namely, for the case $\ell _1=1$ and $\ell _2$ arbitrary).

In the formal identity

$$\begin{aligned}  &   (d-b)(1-aK)(1-cK) + (a-d)(1-bK)(1-cK) \\  &   \qquad + (b-c)(1-aK)(1-dK) + (c-a)(1-bK)(1-dK) = 0, \end{aligned}$$

multiply both sides by

$$\begin{aligned} \frac{(a,b,c,d;q)_k}{e\cdot (qe;q)_k^4}\,q^k \end{aligned}$$

and replace $K\rightarrow q^k$, $a\rightarrow q^{\ell _1}a$, $b\rightarrow q^{-\ell _1}a$, $c\rightarrow q^{\ell _2}a$, $d\rightarrow q^{-\ell _2}a$, $e\rightarrow a$ to get

$$\begin{aligned}&(q^{-\ell _2}-q^{-\ell _1})(1-q^{\ell _1}a)(1-q^{\ell _2}a) f_k(a,q;\ell _1+1,\ell _2+1)\\&\qquad + (q^{\ell _1}-q^{-\ell _2})(1-q^{-\ell _1}a)(1-q^{\ell _2}a) f_k(a,q;\ell _1,\ell _2+1)\\&\qquad + (q^{-\ell _1}-q^{\ell _2})(1-q^{\ell _1}a)(1-q^{-\ell _2}a) f_k(a,q;\ell _1+1,\ell _2)\\&\qquad + (q^{\ell _2}-q^{\ell _1})(1-q^{-\ell _1}a)(1-q^{-\ell _2}a) f_k(a,q;\ell _1,\ell _2) = 0. \end{aligned}$$

Setting $a=1$ in the result, multiplying both sides by $G(a,q;\ell _1,\ell _2)$ and using

$$\begin{aligned} G(a,q;\ell _1+1,\ell _2) =\frac{a-q^{\ell _1}}{1-q^{\ell _1}a}\,G(a,q;\ell _1,\ell _2) \end{aligned}$$

and

$$\begin{aligned} G(a,q;\ell _1,\ell _2+1) =\frac{a-q^{\ell _2}}{1-q^{\ell _2}a}\,G(a,q;\ell _1,\ell _2) \end{aligned}$$

for the product in (2), we find out that

$$\begin{aligned}&(q^{-\ell _2}-q^{-\ell _1})(1-q^{\ell _1}a)(1-q^{\ell _2}a) g_k(a,q;\ell _1+1,\ell _2+1)\\&\qquad + (q^{\ell _1}-q^{-\ell _2})(1-q^{-\ell _1}a)(1-q^{\ell _2}a) g_k(a,q;\ell _1,\ell _2+1)\\&\qquad + (q^{-\ell _1}-q^{\ell _2})(1-q^{\ell _1}a)(1-q^{-\ell _2}a) g_k(a,q;\ell _1+1,\ell _2)\\&\qquad + (q^{\ell _2}-q^{\ell _1})(1-q^{-\ell _1}a)(1-q^{-\ell _2}a) g_k(a,q;\ell _1,\ell _2) = 0, \end{aligned}$$

where

$$\begin{aligned} g_k(a,q;\ell _1,\ell _2) = G(a,q;\ell _1,\ell _2) f_k(1,q;\ell _1,\ell _2). \end{aligned}$$

In particular, both sums

$$\begin{aligned} F_n(a,q;\ell _1,\ell _2) = \sum _{k=0}^{n-1} f_k(a,q;\ell _1,\ell _2) \end{aligned}$$

and

$$\begin{aligned} \sum _{k=0}^{n-1} g_k(a,q;\ell _1,\ell _2) = G(1,q;\ell _1,\ell _2) F_n(1,q;\ell _1,\ell _2) \end{aligned}$$

satisfy the *same* recursion

5 $$\begin{aligned}&(q^{-\ell _2}-q^{-\ell _1})(1-q^{\ell _1}a)(1-q^{\ell _2}a) F(a,q;\ell _1+1,\ell _2+1) \nonumber \\&\qquad + (q^{\ell _1}-q^{-\ell _2})(1-q^{-\ell _1}a)(1-q^{\ell _2}a) F(a,q;\ell _1,\ell _2+1) \nonumber \\&\qquad + (q^{-\ell _1}-q^{\ell _2})(1-q^{\ell _1}a)(1-q^{-\ell _2}a) F(a,q;\ell _1+1,\ell _2) \nonumber \\&\qquad + (q^{\ell _2}-q^{\ell _1})(1-q^{-\ell _1}a)(1-q^{-\ell _2}a) F(a,q;\ell _1,\ell _2) = 0. \end{aligned}$$

Note also that the second sum is shorter when $0<\ell _1,\ell _2<n$:

6 $$\begin{aligned} \sum _{k=0}^{n-1} g_k(a,q;\ell _1,\ell _2) = G(a,q;\ell _1,\ell _2) \sum _{k=0}^{\min \{\ell _1,\ell _2\}-1} \frac{(q^{\ell _1},q^{1-\ell _1},q^{\ell _2},q^{1-\ell _2};q)_k}{(q;q)_k^4}\,q^k. \end{aligned}$$

Furthermore observe that the difference equation in (5) degenerates when $\ell _1=\ell _2$ (and therefore does not give access to comparison of the two solutions along the diagonal $\ell _1=\ell _2$).

In the case $\ell _1=\ell _2$ the difference equation is three-term but less friendly. For simplicity we write $\ell$ instead of $\ell _1=\ell _2$. The announced difference equation corresponds to the difference operator

$$\begin{aligned} \mathcal {L}_q&:= - qL^2(1+L)(1+4L+L^2)(1-qL)^3(1-La)^2(1-qLa)^2 S^2\\&\quad + (1-qL^2) (1 + 5(1+q)L + (5+16q+5q^2)L^2 + (1+q)(1-5q+q^2)L^3\\&\quad - 2q(3+25q+3q^2)L^4 + q(1+q)(1-5q+q^2)L^5 + q^2(5+16q+5q^2)L^6\\&\quad + 5q^3(1+q)L^7 + q^4L^8) (1-La)^2(a-qL)^2 S\\&\quad - qL^2(1-L)^3(1+qL)(1+4qL+q^2L^2)(a-L)^2(a-qL)^2, \end{aligned}$$

where *S* denotes the shift operator $\ell \mapsto \ell +1$, while $L=q^\ell$. Explicitly, if we write $\tilde{f}_k(a,q;\ell )=s(a,q,q^\ell ,q^ka) f_k(a,q;\ell ,\ell )$ with

$$\begin{aligned} s(a,q,L,K)&=\frac{q(1+L)(1+qL)(1-qL^2)(a-L)^2(a-qL)^2L^2(1-K)^4}{K(K-qL)^2(K-L)^2}\\&\quad \times (K(1+q^3L^6)+4K(1+q)(1+q^2L^4)L\\&\quad -(4q^2-K-13qK-q^2K+4K^2)(1+qL^2)L^2\\&\quad -2(q^3+7q(q+K^2)+K^2)L^3), \end{aligned}$$

then^1^

$$\begin{aligned} \mathcal {L}_qf_k(a,q;\ell ,\ell )=\tilde{f}_{k+1}(a,q;\ell )-\tilde{f}_k(a,q;\ell ). \end{aligned}$$

Since $\tilde{f}_n(a,\zeta ;\ell )=\tilde{f}_0(a,\zeta ;\ell )$ for a primitive *n*th root of unity, we find by telescoping that $\mathcal {L}_\zeta F_n(a,\zeta ;\ell ,\ell )=0$. Because of the special shape of the difference operator $\mathcal {L}_q$ and of the certificate *s*(*a*, *q*, *L*, *K*), similarly to the situation where $\ell _1\ne \ell _2$ we conclude that also

$$\begin{aligned} \mathcal {L}_\zeta \big (G(a,\zeta ;\ell ,\ell ) F_n(1,\zeta ;\ell ,\ell )\big )=0. \end{aligned}$$

To prove the identity in Theorem 1, we are going to check it for $\ell _1=\ell _2=0$ and for $\ell _1=1$, $\ell _2$ arbitrary, see below. Assuming this for the moment, by the symmetry $\ell _1\leftrightarrow \ell _2$ the identity then also holds for $\ell _1$ arbitrary and $\ell _2=1$. The cases $\ell _1=\ell _2=0$ and $\ell _1=\ell _2=1$ and the homogeneous recursion associated with the operator $\mathcal {L}_\zeta$ implies that (3) holds when $\ell _1=\ell _2$ is arbitrary. Then the other difference equation implies the validity of (3) for general $\ell _1$ and $\ell _2$.

Now, by definition, we have

$$\begin{aligned} G(a,\zeta ;0,0) =G(a,\zeta ;1,1)=1. \end{aligned}$$

At the same time, the equality $F_n(a,\zeta ;0,0)=F_n(a,\zeta ;1,1)$ follows from the definition (1). On the basis of the three-term recursion satisfied by $F_n(a,\zeta ;\ell ,\ell )$ and by $G(a,\zeta ;\ell ,\ell ) F_n(1,\zeta ;\ell ,\ell )$ this implies the proportionality of the two for every $\ell \in \mathbb Z$.

When $\ell _1=1$, the sum $\sum _k g_k(a,\zeta ;1,\ell _2)$ reduces to the single entry for $k=0$ (see (6)). Thus, this case requires the verification of

7 $$\begin{aligned} \sum _{k=0}^{n-1} \frac{(1-a)\,(\zeta ^{\ell }a,\zeta ^{1-\ell }a;\zeta )_k}{(1-\zeta ^ka)\,(\zeta a;\zeta )_k^2}\,\zeta ^k&=\frac{n^2a^{n-1}}{(1+a+a^2+\dots +a^{n-1})^2}\,G(a,\zeta ;1,\ell ) \nonumber \\&=\frac{n^2a^{n-1}}{(1+a+a^2+\dots +a^{n-1})^2}\,\frac{\prod _{j=1}^{\ell -1}(a-\zeta ^j)}{\prod _{j=1}^{\ell -1}(1-\zeta ^ja)}. \end{aligned}$$

We now take

$$\begin{aligned} h_k(a,q;\ell )=\frac{(1-a)\,(q^{\ell }a,q^{1-\ell }a;q)_k}{(1-q^ka)\,(qa;q)_k^2}\,q^k \end{aligned}$$

and

$$\begin{aligned} \tilde{h}_k(a,q;\ell )&=\frac{(1-q^ka)^3(1+q^\ell )(a-q^\ell )q^{\ell -k}}{a\,(1-q^\ell )^2(q^\ell -q^ka)}\,h_k(a,q;\ell ) \end{aligned}$$

with the motive that

$$\begin{aligned} (1-q^\ell a)h_k(a,q;\ell +1) -(a-q^\ell )h_k(a,q;\ell ) =\tilde{h}_{k+1}(a,q;\ell )-\tilde{h}_k(a,q;\ell ). \end{aligned}$$

Substituting $q=\zeta$ and summing over *k* from 0 to $n-1$ we obtain

$$\begin{aligned} (1-\zeta ^\ell a)H(a,\zeta ;\ell +1) -(a-\zeta ^\ell )H(a,\zeta ;\ell ) =\tilde{h}_n(a,\zeta ;\ell )-\tilde{h}_0(a,\zeta ;\ell )=0, \end{aligned}$$

where

$$\begin{aligned} H(a,\zeta ;\ell )=\sum _{k=0}^{n-1}h_k(a,\zeta ;\ell ) =\sum _{k=0}^{n-1} \frac{(1-a)\,(\zeta ^{\ell }a,\zeta ^{1-\ell }a;\zeta )_k}{(1-\zeta ^ka)\,(\zeta a;\zeta )_k^2}\,\zeta ^k. \end{aligned}$$

This shows that verification in (7) reduces to the one where $\ell =1$, that is, to

$$\begin{aligned} \sum _{k=0}^{n-1} \frac{1}{(1-\zeta ^ka)^2}\,\zeta ^k =\frac{n^2a^{n-1}}{(1-a^n)^2} =\frac{n^2a^{n-1}}{\prod _{j=0}^{n-1}(1-\zeta ^ja)^2}. \end{aligned}$$

The latter sum on the left-hand side is the partial fraction decomposition of the right-hand side.

This shows in particular that the identity (3) holds for $\ell _1=\ell _2=1$. By what we have already seen, the validity of (3) follows for $\ell _1=\ell _2$ arbitrary.

## Footnotes

1 We found this creative telescoping relation using the implementation of the *q*-analogue of Zeilberger’s algorithm (cf. [3, 5]) within the QDifferenceEquations package in *Maple*.

## References

[1] Garoufalidis, S., Zagier, D.: Asymptotics of Nahm sums at roots of unity. Ramanujan J. **55**, 219–238 (2021)

[2] Kashaev, R.M., Mangazeev, V.V., Stroganov, Y.G.: Star-square and tetrahedron equations in the Baxter-Bazhanov model. Int. J. Mod. Phys. A **8**(8), 1399–1409 (1993)

[3] Koornwinder, T.H.: On Zeilberger’s algorithm and its -analogue. J. Comput. Appl. Math. **48**, 91–111 (1993)

[4] Spiridonov, V., Zhedanov, A.: Zeros and orthogonality of the Askey-Wilson polynomials for a root of unity. Duke Math. J. **89**, 283–305 (1997)

[5] Zeilberger, D.: A fast algorithm for proving terminating hypergeometric identities. Discrete Math. **80**(2), 207–211 (1990)

[6] Zudilin, W.: (-)Supercongruences hit again. Hardy-Ramanujan J. **43**, 46–55 (2020)

